# Supplementary material for: Dynamic Changes in Flavor and Microbiota in Traditionally Fermented Bamboo Shoots (Chimonobambusa szechuanensis (Rendle) Keng f.)
Source: Foods. 2023 Aug 12;12(16):3035. doi: 10.3390/foods12163035 (PMC10453856; doi:10.3390/foods12163035)
Supplement: Supplementary file 1 [file foods-12-03035-s001.zip › foods-2521262-supplementary.pdf]

Supplementary data

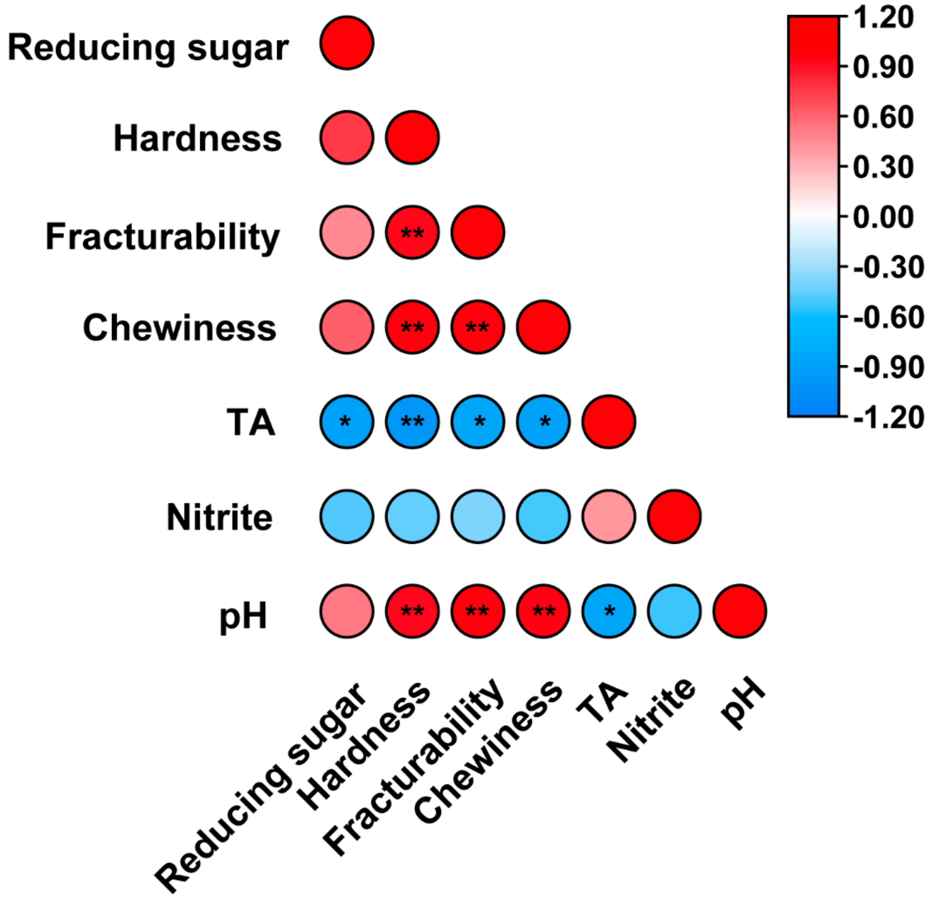

Figure S1 Correlation among reducing sugar, hardness, fracturability, chewiness, TA, pH, and nitrite of sour bamboo shoots conducted by Pearson's correlation analysis. Circles represent a positive (orange) or negative (blue) correlation between the quality indicators. The size of the circle represents the levels of the correlation coefficient (r), * $P < 0.05$, ** $P < 0.01$.

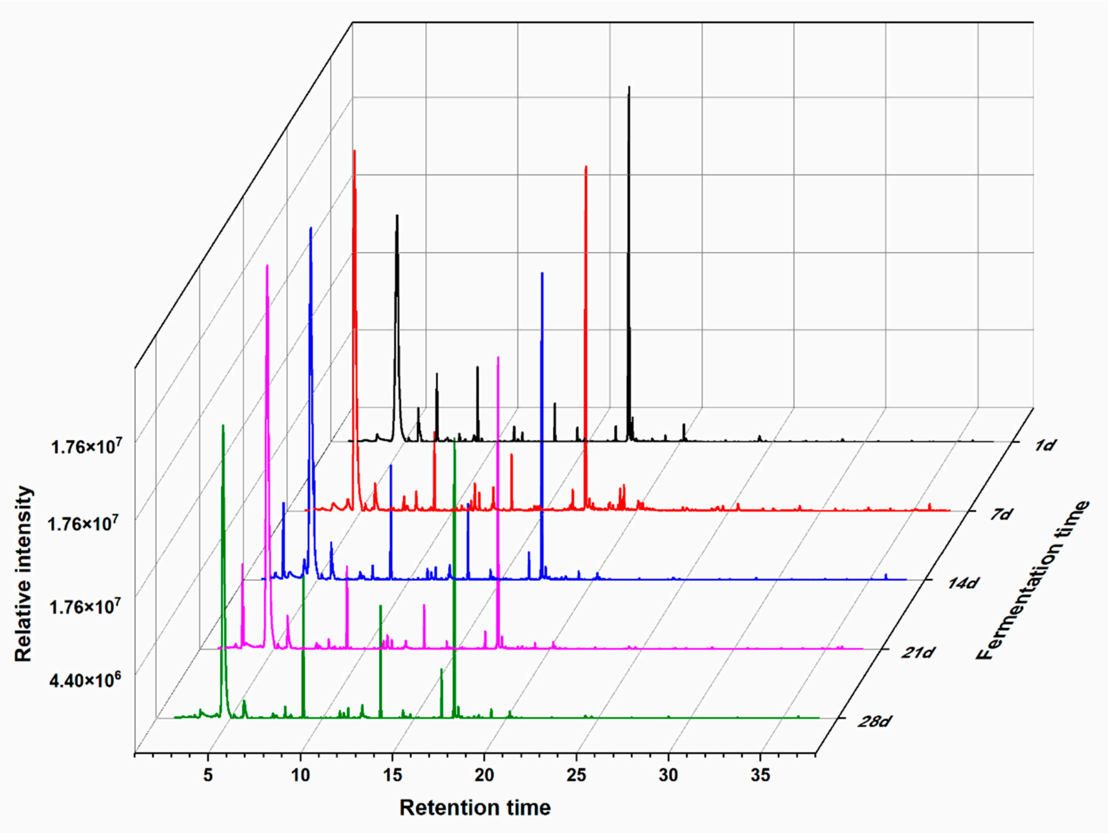

Figure S2 Total ion flow of volatile compounds in sour bamboo shoots during the fermentation process.

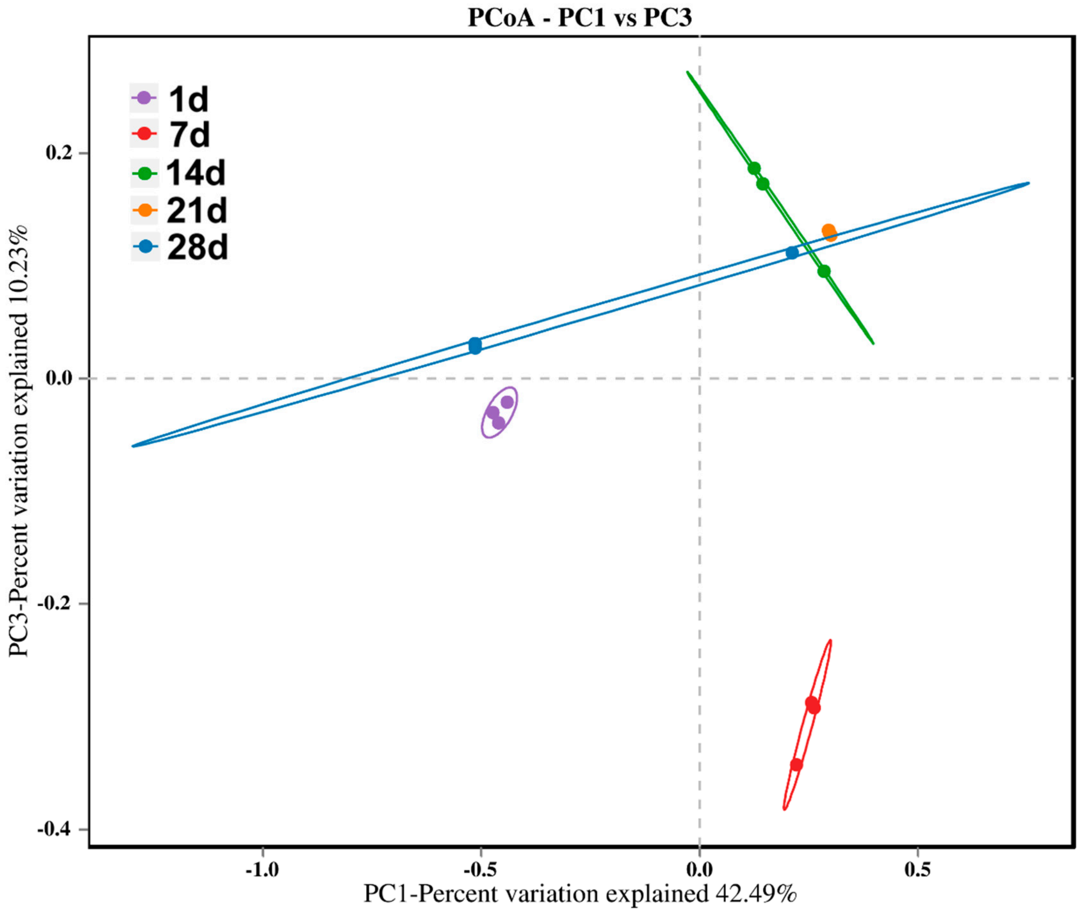

Figure S3 PCoA analysis of the microbial community in sour bamboo shoots during fermentation process.

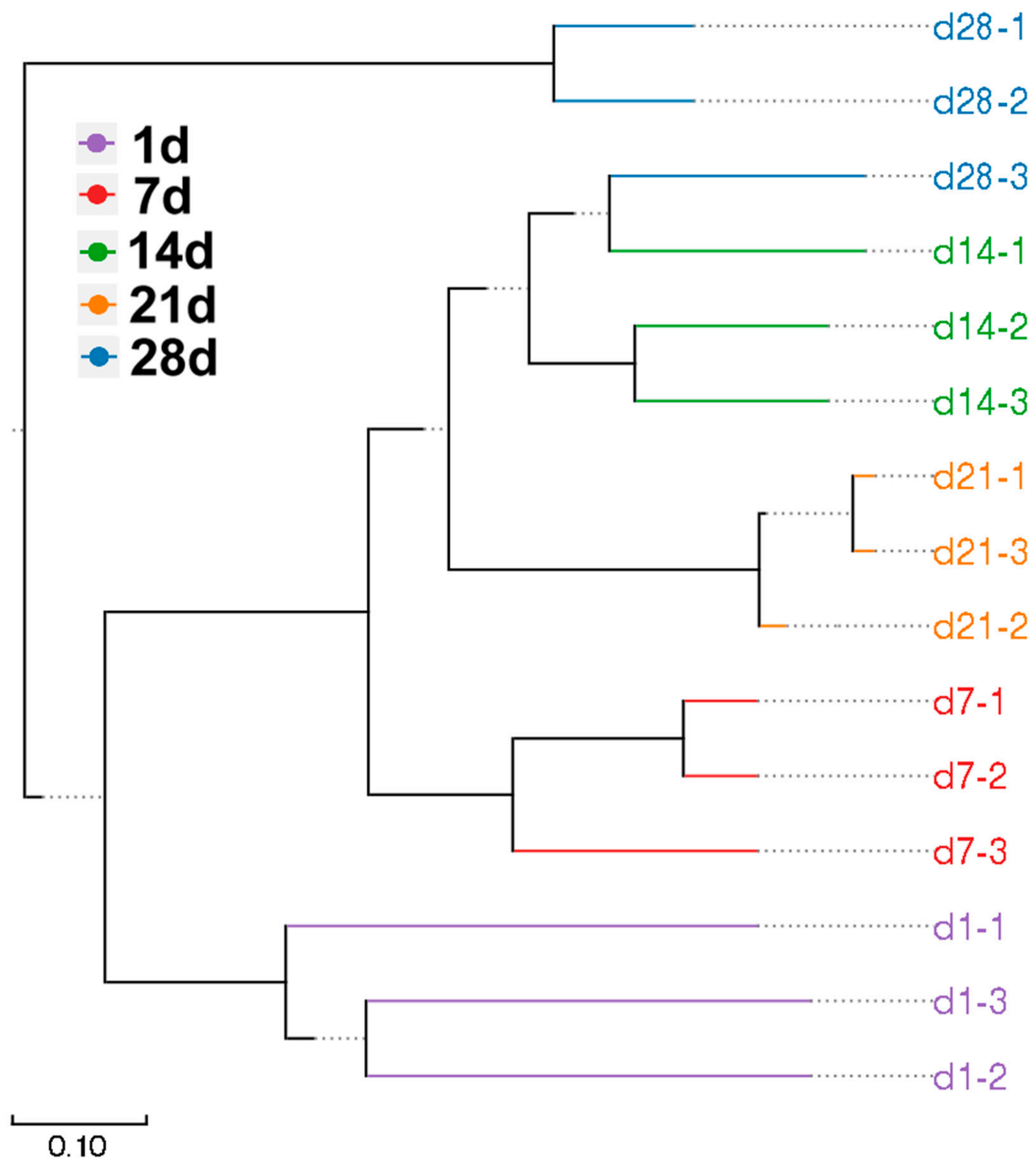

Figure S4 UPGMA analysis of the microbial community in sour bamboo shoots during fermentation process.

Table S1 Volatile compounds of sour bamboo shoots during fermentation measure by GC-MS.

NO.	Volatile components	Relative content (μg/L)				
		1d	7d	14d	21d	28d
Alcohols						
1	1-Heptanol	-	-	8.05±0.77b	-	-
2	1-Octen-3-ol	101.10 ±0.00b	-	-	-	117.68±10.33a
3	2-Ethyl-1-hexanol	50.54 ±0.00a	14.40±1.13b	8.07±0.72d	7.20±1.85d	-
4	(E)-2-Octen-1-ol	8.98±12.70a	18.63±2.06a	14.43±1.26a	13.77±3.38a	11.55±1.75a
5	1-Octanol	-	-	-	-	14.06±3.91a
6	Linalool	1.36±1.92c	-	4.13±0.34c	4.36±0.10c	8.96±0.00b
7	3,6-Nonadien-1-ol	0.84±1.18e	104.85±5.34b	76.60±6.16cd	69.56±4.52d	87.49±2.39c
8	1-Decanol	-	2.88±0.39a	1.43±0.32b	1.90±0.20	3.84±0.34a
9	1-Pentanol	74.48±0.00a	18.26±1.10b	-	-	-
10	dimethylsilanediol	1.74 ±0.00c	-	317.50±58.65b	74.05±33.15a	422.08±0.00a
11	1-Nonanol	2.01 ±0.15c	6.10±0.51a	3.09±0.18b	3.07±0.42b	2.69±0.58bc
12	1-Dodecanol	2.43 ±0.00b	9.77±0.64a	2.16±0.33b	1.56±0.11b	2.49±0.69b
13	3-Methyl-1-butanol	14.85 ±0.00c	23.66±1.44b	-	-	-
14	Cyclooctyl alcohol	8.99 ±0.00b	-	-	-	-
15	Cedrol	3.10 ±0.00c	13.99±0.92a	2.28±0.34c	2.55±0.72c	2.61±0.65c
16	Cyclohexanol, 2-methyl-	-	7.70±0.35a	1.37±0.00b	-	-
17	Bicyclo[3.1.1]heptane-2-methanol,6,6-dimethyl-, (1S,2S,5S)-	-	-	-	-	5.49±0.00b
Esters						
18	2,2,4-Trimethyl-1,3-pentanediol diisobutyrate	7.74±3.32c	25.65±3.34a	17.14±0.16b	14.73±1.50bc	14.45±1.96bc

19	Propanoic acid, 2-methyl-, 3-hydroxy-2,2,4-trimethylpentyl ester	3.42±1.43c	18.70±1.13a	7.83±1.82b	3.94±0.32c	5.16±0.53bc
20	Hexanoic acid, 3,5,5-trimethyl-, 2-ethylhexyl ester	9.94±0.72b	17.86±0.80a	8.80±1.21b	4.34±0.31c	8.25±0.85b
21	1,2-Benzenedicarboxylic acid, bis(2-methylpropyl) ester	17.74±11.54bc	22.24±0.58b	50.35±2.61a	10.96±2.22c	25.54±0.68b
22	Dibutyl phthalate	15.36±10.21b	13.24±0.84b	25.04±0.00a	13.69±0.00b	8.38±0.00b
23	Formic acid, octyl ester	-	21.83±1.34a	15.08±0.82b	11.41±0.92c	-
24	Hexadecanoic acid, methyl ester	-	-	-	-	2.27±0.00b
Aldehydes						
25	Octanal	2.50±1.31b	5.27±0.38a	1.56±0.28b	1.43±0.22b	2.32±0.12b
26	Nonanal	9.62±4.01c	16.30±0.11ab	8.76±1.29c	19.36±0.57a	17.26±1.78a
27	Decanal	5.37±0.75bc	13.11±1.01a	4.21±0.13cd	3.41±0.00d	4.27±0.20cd
28	Benzaldehyde, 2,4-dimethyl-	25.95±3.87d	133.77±7.15a	39.74±4.13c	69.28±2.71b	33.41±1.58cd
29	Dodecanal	0.98±0.37d	5.95±0.33a	2.60±0.19b	2.09±0.30c	1.22±0.06d
Phenols						
30	2,4-Di-tert-butylphenol	2249.37±236.93a	1353.07±70.59e	1417.76±73.07de	1692.12±65.90cd	1939.50±143.94bd
31	2-Methoxy-4-vinylphenol	-	4.94±0.26c	-	6.19±0.22c	36.95±0.00a
32	Phenol, 2,5-bis(1,1-dimethylethyl	-	2.49±0.00c	2.48±0.00d	2.42±0.00e	4.50±0.00a
Ketones						
33	Isophorone	70.16±15.37b	21.06±0.86c	-	-	7.82±0.00d
34	3-Hepten-2-one, 5-ethyl-6-methyl-	13.55±2.00a	6.29±0.59bc	5.17±0.47bc	-	4.85±0.67c
35	2,5-Cyclohexadien-1-one, 2,6-bis(1,1-dimethylethyl)-4-hydroxy-4-methyl-	4.27±1.28b	6.62±0.48a	3.48±0.29b	-	6.45±0.44a
36	2,5-Cyclohexadiene-1,4-dione, 2,6-bis(1,1-dimethylethyl)-	4.82±0.34b	6.25±0.27a	2.97±0.13c	-	2.89±0.05c
37	7,9-ditert-butyl-1-oxaspiro[4.5]deca-6,9-diene-2,8-dione	13.45±7.38a	-	-	-	0.10±0.00b
38	Cyclohexanone, 2-cyclohexylidene-	1.29±0.00bc	5.92±0.02a	3.26±0.88abc	-	4.69±0.13ab
Others						
39	D-Limonene	354.21±33.00a	10.45±0.38b	2.41±0.45b	-	3.22±0.09b
40	Naphthalene	5.13±0.35cd	25.45±2.02a	7.78±0.00c	5.71±0.74cd	2.98±0.32d

41	p-Xylene	-	139.10±4.38b	215.11±4.17a	-	37.43±2.36c
42	Benzene, 1,3-dimethyl-	-	2.72±0.75e	5.62±0.82d	6.49±0.00c	8.54±0.27a
Acids						
43	Acetic acid	-	-	31.32±9.79	-	-

-: not detected.

Different letters in same row indicate significant differences between groups ($P < 0.05$).

Table S2 Alpha diversity of the microbial community in sour bamboo shoots during the fermentation process.

	Chao1	ACE	Shannon	Simpson	Coverage
1d	1454.07 ± 269.71a	1454.82 ± 269.79a	9.06 ± 0.28a	0.99 ± 0.00a	0.99
7d	444.67 ± 52.85b	444.89 ± 52.96b	4.34 ± 0.15b	0.88 ± 0.01b	1.0
14d	473.38 ± 165.44b	473.48 ± 165.49b	4.71 ± 0.67b	0.88 ± 0.02b	1.0
21d	141.04 ± 11.02c	141.50 ± 10.96c	3.66 ± 0.03c	0.86 ± 0.00b	1.0
28d	493.35 ± 78.11b	493.44 ± 78.20b	2.87 ± 1.03d	0.57 ± 0.17c	1.0
